# Supplementary material for: The audio features of sleep music: Universal and subgroup characteristics
Source: PLoS One. 2023 Jan 18;18(1):e0278813. doi: 10.1371/journal.pone.0278813 (PMC9847986; doi:10.1371/journal.pone.0278813)
Supplement: S1 Table — (DOCX) [file pone.0278813.s001.docx]

| **S1 Table:** Playlist exclusion criteria | | |
| --- | --- | --- |
| Playlist exclusion criteria | Description | Occurrences |
| Non-music | Rain sounds | 29 |
|  | Ocean/water sounds | 26 |
|  | Other | 23 |
|  | Noise | 18 |
|  | Thunder/storm sounds | 15 |
|  | Nature sounds | 13 |
|  | ASMR | 10 |
|  | Night/Sleep sounds | 10 |
|  | Guided meditation | 9 |
|  | Continuous tones | 8 |
|  | Hypnosis | 6 |
|  | Stories | 5 |
|  | Reading of religious texts | 5 |
|  | ‘Learn a language while sleeping’ | 4 |
|  | Affirmations | 4 |
|  | Podcasts | 3 |
|  | Whale sounds | 2 |
| Non-sleep | Sleep means something else | 20 |
|  | Other purpose than to sleep | 13 |
|  | Negative word before the word sleep | 9 |
| Non-human | Playlists meant for dogs | 12 |
| Non-representative | Fewer than 100 followers | 33 |
| *Note:* ‘Other’ includes *Fan Sounds, Hoover Sounds, Fire Sounds, Vaporwave Sleep, Train Sounds, etc.* ‘Sleep means something else’ refers to an instance in which the word sleep means something other than the act of going to sleep, such as a band called “The Sleepy Sleepers” or a soundtrack of the film “Before I go to sleep” and “Sleepless in Seattle”. | | |
