# Supplementary material for: The audio features of sleep music: Universal and subgroup characteristics
Source: PLoS One. 2023 Jan 18;18(1):e0278813. doi: 10.1371/journal.pone.0278813 (PMC9847986; doi:10.1371/journal.pone.0278813)
Supplement: S2 Table — (DOCX) [file pone.0278813.s002.docx]

| **S2 Table**: Descriptive statistics of the Sleep Playlist Dataset | | | | | | |
| --- | --- | --- | --- | --- | --- | --- |
|  | Minimum | 1^st^ Quartile | Median | Mean | 3^rd^ Quartile | Maximum |
| Duration of playlist (minutes) | 15 | 601 | 1429 | 6799 | 19210 | 21889 |
| Number of followers per playlist | 102 | 879 | 1,932 | 25,833 | 8,528 | 3,982,105 |
| Duration of track (minutes) | 0.1 | 2.04 | 2.56 | 3.07 | 3.6 | 97.11 |
| Number of tracks per playlist | 2 | 187 | 434 | 2956 | 8350 | 9991 |
