# Supplementary material for: The audio features of sleep music: Universal and subgroup characteristics
Source: PLoS One. 2023 Jan 18;18(1):e0278813. doi: 10.1371/journal.pone.0278813 (PMC9847986; doi:10.1371/journal.pone.0278813)
Supplement: S3 Table — (DOCX) [file pone.0278813.s003.docx]

| Cluster 6  N= 5783 | Cluster 5  N= 32 651 | Cluster 4  N= 117 237 | Cluster 3  N= 30 721 | Cluster 2  N= 30 959 | Cluster 1  N= 8 275 | Cluster Number | **S3 Table:** Most frequent musical genres, audio features and tracks based on trackID of the 6 clusters |
| --- | --- | --- | --- | --- | --- | --- | --- |
| Live Tracks | Instrumental Tracks | Ambient Tracks | Acoustic Radio Tracks | Radio Tracks | Speechy Tracks | Cluster Name |  |
| High liveness | High instrumentalness,  high acousticness,  high danceability | High acousticness,  high instrumentalness,  low danceability | High energy,  high danceability,  high acousticness, | High energy,  high danceability,  low acousticness, | High speechiness | Most prominent musical feature(s) |  |
| Jazz,  Lofi,  Guitar,  Piano,  Classical | Lofi,  Ambient,  Classical,  Lullaby,  Christian | Lofi,  R&B,  Rap,  Pop | Pop,  Lofi,  R&B,  Rap,  Kpop,  Country,  Indie | Pop,  Indie,  Lofi,  Jazz | Ambient,  Relaxation | Most frequent genre |  |
| Death bed (coffee in your head)  The Night We Met  Will He  Always Remember Us This Way  All the kids are depressed | everything i wanted  5:32pm  this girl  I’m in love with you, sorry  I’ll keep you safe | 936Hz Courage & Inner Power  936Hz Activate Pineal Gland  936Hz Remove Self Limiting Beliefs  936  Solfeggio Frequencies 936 | Jealous  lovely (with Khalid)  Falling  i love you  The Scientist | Dynamite  Fix You  Perfect  Your Eyes Tell  How To Save A Life | Jocelyn Flores  I hate you, I love you  I don’t sleep  Getsomerest/sleepwell  Falling for U | Top 5 tracks of each cluster based on trackID |  |
| – Powfu  – Lord Huron  – Joji  – Lady Gaga  – Jeremy Zucker | – Billie Eilish  – The Vibes  – Wlijah Who  – j’san  – sagun | – Miracle Tones  – Miracle Tones  – Miracle Tones  – Timo Krantz  – Miracle Tones | – Labyrinth  – Billie Eilish  – Harry Styles  – Billie Eilish  – Coldplay | – BTS  – Coldplay  – Ed Sheeran  – BTS  – The Fray | – XXTENTACION  – gnash, Olivia O’Brian  – Sarcastic Sounds  – Quickly, quickly  – Peachy! | Artist |  |
