# Supplementary material for: The audio features of sleep music: Universal and subgroup characteristics
Source: PLoS One. 2023 Jan 18;18(1):e0278813. doi: 10.1371/journal.pone.0278813 (PMC9847986; doi:10.1371/journal.pone.0278813)
Supplement: S4 Table — (DOCX) [file pone.0278813.s004.docx]

| **S4 Table**: Top 20 most frequent tracks based on trackID | | | |
| --- | --- | --- | --- |
| Order | Frequency | Track title | Artist |
| 1 | 245 | Dynamite | BTS |
| 2 | 62 | Jealous | Labrinth |
| 3 | 60 | lovely (with Khalid) | Billie Eilish |
| 4 | 57 | Falling | Harry Styles |
| 5 | 54 | i love you | Billie Eilish |
| 6 | 52 | The Scientist | Coldplay |
| 7 | 50 | Supermarket Flowers | Ed Sheeran |
| 8 | 49 | Say You Won’t Let Go | James Arthur |
| 9 | 49 | Someone You Loved | Lewis Capaldi |
| 10 | 48 | when the party’s over | Billie Eilish |
| 11 | 48 | Not About Angels | Birdy |
| 12 | 46 | Happier | Ed Sheeran |
| 13 | 45 | everything i wanted | Billie Eilish |
| 14 | 45 | 5:32pm | The Deli |
| 15 | 44 | Get You The Moon (ft Snow) | Kina |
| 16 | 43 | Fix You | Coldplay |
| 17 | 42 | Perfect | Ed Sheeran |
| 18 | 42 | Dancing On My Own | Calum Scott |
| 19 | 42 | Your Eyes Tell | BTS |
| 20 | 42 | Skinny Love | Birdy |
