# Supplementary material for: The audio features of sleep music: Universal and subgroup characteristics
Source: PLoS One. 2023 Jan 18;18(1):e0278813. doi: 10.1371/journal.pone.0278813 (PMC9847986; doi:10.1371/journal.pone.0278813)
Supplement: Attachment — Submitted filename: Review PONE.pdf [file pone.0278813.s005.pdf]

Authors measured the characteristics of music associated with sleep by extracting audio features from a large number of tracks (N = 225,626) retrieved from sleep playlists at the global streaming platform Spotify. The results suggested that sleep music is characterised by lower Tempo, Loudness, Energy and Tempo and is more likely to have high Instrumentalness and Acousticness values than general music. However, even within sleep music, a large variation of music features remains. The large variation described by the six subgroups authors identified based on their audio features. As the authors stated the findings reveal previously unknown aspects of sleep music and highlight the individual variation in the choice of music for facilitating sleep.

In general, the article is well-written and the authors presented their results in a proper way. I have just some minor comments about the manuscript.

1. I would recommend to clarify the importance of the study and add some practical implication e.g. how music therapist get help to find music to their patients with sleep problems and/or how the findings can be helpful for researchers who are planning experimental studies to understand more about the connections between listening to music and sleep quality.
2. I suggest to write an independent section about the limitation of the study (maybe authors can include here what they mentioned in the supplementary discussion)
3. One specific comment about the novelty of the study, i.e. the results suggested that sleep music is characterised by lower Tempo, Loudness, Energy than general music but people also use music with high Energy, and Danceability would be counterproductive for relaxation and sleep. In one hand, I agree with the authors this type of genre of music could increase relaxation when considering the interplay between repeated exposure, familiarity and predictive processing. In other hand, not only high Energy and Danceability but faster tempo appeared also in the first three cluster can be counterproductive with relaxation effect and sleep according to the entrainment of autonomous biological oscillators such as respiration and heart rate to external stimuli like the beat of the music. I am not sure only familiarity of the music overcome all of these effects may increase physiological arousal however a music with fast tempo and highly repetitive rhythm does not vary throughout of the piece, may help to create a hypnotic feel in the listeners and it has a sleep inducing effect.

Mazzarolo, I. (2019). Infant Sleep through Noise and Music.

Scarratt, R. J., Heggli, O. A., Vuust, P., & Jespersen, K. V. (2021). The music that people use to sleep: universal and subgroup characteristics.
