# Supplementary material for: The audio features of sleep music: Universal and subgroup characteristics
Source: PLoS One. 2023 Jan 18;18(1):e0278813. doi: 10.1371/journal.pone.0278813 (PMC9847986; doi:10.1371/journal.pone.0278813)
Supplement: Attachment — Submitted filename: Response to reviewers.docx [file pone.0278813.s006.docx]

PONE-D-22-01555

The music that people use to sleep: universal and subgroup characteristics

PLOS ONE

New title: The audio features of sleep music: universal and subgroup characteristics

**Journal Requirements:**

When submitting your revision, we need you to address these additional requirements.

1. Please ensure that your manuscript meets PLOS ONE's style requirements, including those for file naming. The PLOS ONE style templates can be found at

<https://journals.plos.org/plosone/s/file?id=wjVg/PLOSOne_formatting_sample_main_body.pdf> and

<https://journals.plos.org/plosone/s/file?id=ba62/PLOSOne_formatting_sample_title_authors_affiliations.pdf>

Thanks for the guidelines. We have edited the manuscript to ensure that it meets PLOS ONE’s style requirements.

2. In your Methods section, please include additional information about your dataset and ensure that you have included a statement specifying whether the collection and analysis method complied with the terms and conditions for the source of the data.

Thank you. We have included a sentence stating that we did not collect personal information and that we comply with Spotify’s terms of use. Please see p. 6 ll. 119-121.

3. Thank you for stating the following in the Acknowledgments Section of your manuscript:

“Center for Music in the Brain is funded by the Danish National Research Foundation (DNRF117). The authors received no specific funding for this work.”

We note that you have provided additional information within the Acknowledgements Section that is not currently declared in your Funding Statement. Please note that funding information should not appear in the Acknowledgments section or other areas of your manuscript. We will only publish funding information present in the Funding Statement section of the online submission form.

Please remove any funding-related text from the manuscript and let us know how you would like to update your Funding Statement. Currently, your Funding Statement reads as follows:

“The author(s) received no specific funding for this work.”

Please include your amended statements within your cover letter; we will change the online submission form on your behalf.

Thanks for this clarification. We have removed the text from the Acknowledgements section and would like you to kindly update the Funding Statement to “Center for Music in the Brain is funded by the Danish National Research Foundation (DNRF117). The authors received no specific funding for this work.”. This is also included in the cover letter.

**Reviewers' comments:**

Reviewer #1:

1. The title of the paper promises that the study is about music to be used to sleep. Unfortunately this is not the case, because the study does not investigate whether the music really causes sleep. The study is just what people think that the music might help to sleep. This is a different question. No effects on sleep were investigated. It is just the assumption that people think that the music has a positive effect on sleep. In order to make this clear, I suggest to modify the title of the paper and to modify the abstract as well.

The processing of Spotify music tracks is nice and entertaining. The processing seems to be done in a correct way. However, all this misses the effect on sleep. It could be so, that you search for music which creates good mood or promisses love or peace and no assessment on what the music promisses or what the assumed effect is. The paper is on the analysis of music tracks and this can be a valid analysis, but this is not my area of expertise.

Thank you very much! We have changed the title of the manuscript and edited the abstract to ensure that they clearly reflect the focus of this study.

Reviewer #2:

Authors measured the characteristics of music associated with sleep by extracting audio features from a large number of tracks (N = 225,626) retrieved from sleep playlists at the global streaming platform Spotify. The results suggested that sleep music is characterised by lower Tempo, Loudness, Energy and Tempo and is more likely to have high Instrumentalness and Acousticness values than general music. However, even within sleep music, a large variation of music features remains. The large variation described by the six subgroups authors identified based on their audio features. As the authors stated the findings reveal previously unknown aspects of sleep music and highlight the individual variation in the choice of music for facilitating sleep.
In general, the article is well-written and the authors presented their results in a proper way. I have just some minor comments about the manuscript.

1. I would recommend to clarify the importance of the study and add some practical implication e.g. how music therapist get help to find music to their patients with sleep problems and/or how the findings can be helpful for researchers who are planning experimental studies to understand more about the connections between listening to music and sleep quality.

Thank you very much for these suggestions. In the introduction, we have clarified the contribution of this study, and in the discussion, we have added a section on the relevance of the study to both clinical practice as well as research in the field of music and sleep.

2. I suggest to write an independent section about the limitation of the study (maybe authors can include here what they mentioned in the supplementary discussion)

Thank you. We have added a section on limitations implementing the considerations from the supplementary information.

3. One specific comment about the novelty of the study, i.e. the results suggested that sleep music is characterised by lower Tempo, Loudness, Energy than general music but people also use music with high Energy, and Danceability would be counterproductive for relaxation and sleep. In one hand, I agree with the authors this type of genre of music could increase relaxation when considering the interplay between repeated exposure, familiarity and predictive processing. In other hand, not only high Energy and Danceability but faster tempo appeared also in the first three cluster can be counterproductive with relaxation effect and sleep according to the entrainment of autonomous biological oscillators such as respiration and heart rate to external stimuli like the beat of the music. I am not sure only familiarity of the music overcome all of these effects may increase physiological arousal however a music with fast tempo and highly repetitive rhythm does not vary throughout of the piece, may help to create a hypnotic feel in the listeners, and it has a sleep inducing effect.

Thank you for pointing this out. We agree that familiarity may not be the only factor to explain why people use faster tempo music for sleep, and we have expanded our discussion to consider also the dynamics of each track in terms of repetition and variation within the music track.
